# The frequency and clinical associations of opioid use in systemic sclerosis

**DOI:** 10.1093/rap/rkae144

**Published:** 2024-11-15

**Authors:** Jessica L Fairley, Dylan Hansen, Susanna Proudman, Joanne Sahhar, Gene-Siew Ngian, Diane Apostolopoulos, Jennifer Walker, Lauren V Host, Wendy Stevens, Nava Ferdowsi, Maryam Tabesh, Mandana Nikpour, Laura Ross

**Affiliations:** Department of Medicine at St Vincent’s Hospital Melbourne, University of Melbourne, Melbourne, VIC, Australia; Department of Rheumatology, St Vincent’s Hospital Melbourne, Melbourne, VIC, Australia; Department of Rheumatology, St Vincent’s Hospital Melbourne, Melbourne, VIC, Australia; Department of Medicine, University of Adelaide, Adelaide, SA, Australia; Department of Rheumatology, Royal Adelaide Hospital, Adelaide, SA, Australia; Department of Rheumatology, Monash Health, Melbourne, VIC, Australia; Department of Medicine, Monash University, Melbourne, VIC, Australia; Department of Rheumatology, Monash Health, Melbourne, VIC, Australia; Department of Medicine, Monash University, Melbourne, VIC, Australia; Department of Rheumatology, Monash Health, Melbourne, VIC, Australia; Department of Rheumatology, Royal Adelaide Hospital, Adelaide, SA, Australia; Department of Rheumatology, Fiona Stanley Hospital, Perth, WA, Australia; Department of Rheumatology, St Vincent’s Hospital Melbourne, Melbourne, VIC, Australia; Department of Rheumatology, St Vincent’s Hospital Melbourne, Melbourne, VIC, Australia; Department of Rheumatology, St Vincent’s Hospital Melbourne, Melbourne, VIC, Australia; University of Sydney School of Public Health, University of Sydney, Sydney, NSW, Australia; Department of Rheumatology, Royal Prince Alfred Hospital, Sydney, NSW, Australia; Sydney Musculoskeletal Research Flagship Centre, University of Sydney, Sydney, NSW, Australia; Department of Medicine at St Vincent’s Hospital Melbourne, University of Melbourne, Melbourne, VIC, Australia; Department of Rheumatology, St Vincent’s Hospital Melbourne, Melbourne, VIC, Australia

**Keywords:** opioid, systemic sclerosis, quality of life

## Abstract

**Objective:**

To define the frequency and associations of opioid use in SSc.

**Methods:**

Australian Scleroderma Cohort Study participants meeting ACR/EULAR criteria for SSc were included. Current or previous opioid use was recorded at each visit, with long-term use defined as use on two or more consecutive visits. Groups were compared using two-sample *t*-test, Wilcoxon rank sum test or chi-squared test. Generalised estimating equations were used to model longitudinal data.

**Results:**

Of 1951 participants with a mean age of 46.7 years (s.d. 14.4), 88% were female and 12% had ever received any opioids since SSc onset. Of these, 46% recorded opioid use across multiple consecutive study visits. Digital ulcers (63% *vs* 52%), synovitis (57% *vs* 38%), interstitial lung disease (37% *vs* 27%), gastrointestinal (GI) symptoms (upper 97% *vs* 88%, lower 90% *vs* 80%) and immunosuppression (59% *vs* 46%) were all more frequent in opioid-exposed groups (*P* < 0.05). In multivariable modelling, current opioid use at each study visit was associated with digital ulcers [odds ratio (OR) 1.5 (95% CI 1.1, 2.0), *P* = 0.01], synovitis [OR 1.5 (95% CI 1.1, 2.1), *P* = 0.02], lower GI symptoms [OR 1.8 (95% CI 1.3, 2.6), *P* < 0.01] and poorer physical [OR 1.8 (95% CI 1.3, 2.4), *P* < 0.01] and mental [OR 1.8 (95% CI 1.1, 3.0), *P* = 0.02] quality of life (QoL). Current opioid use was associated with worse fatigue [regression coefficient (RC) 3.0 units (95% CI 1.2, 4.8), *P* < 0.01], functional disability [RC 0.2 (95% CI 0.2, 0.3), *P* < 0.01], dyspnoea [RC 2.0 (95% CI 0.8, 3.1), *P* < 0.01], depression [RC 2.5 (95% CI 0.9, 4.0), *P* < 0.01] and anxiety [RC 2.5 (95% CI 0.9, 4.0), *P* < 0.01].

**Conclusions:**

Opioid use in SSc was associated with musculoskeletal, GI and lung involvement. Opioid prescription was associated with poorer QoL and physical function.

## Graphical Abstract

**Graphical Abstract:**
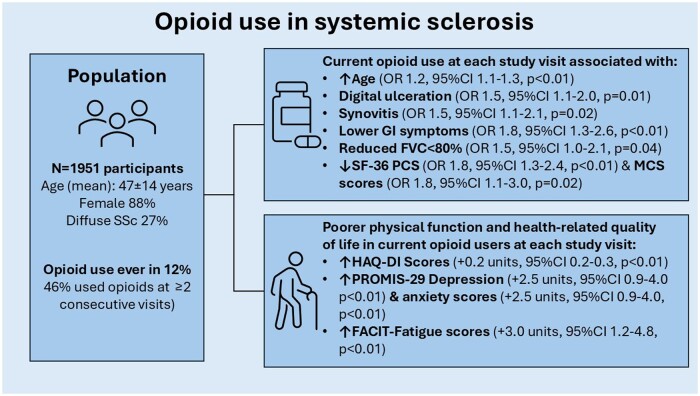

Key messages

- Twelve percent of a large, longitudinal Australian systemic sclerosis cohort used opioids, with 46% using opioids across multiple consecutive study visits.
- Current opioid use was associated with digital ulceration, synovitis, lower gastrointestinal symptoms and impaired spirometry.
- Opioid use was associated with poorer physical function and health-related quality of life.

## Introduction

Up to 60% of people living with an autoimmune rheumatic disease may have a concomitant diagnosis of a chronic pain syndrome [1], which can increase the risk of depression, surgery and hospitalisation [1]. Therefore people with autoimmune rheumatic diseases may be at increased risk of opioid use, with 13–20% of patients with SLE being prescribed opioids [2], as well as 30–40% of patients with inflammatory arthritis, even after biologic initiation [3]. This is in comparison to the general population, where chronic pain is reported in 11.2% of individuals and with 3–4% of the population prescribed long-term opioid prescriptions [4]. Up to 90% of people with SSc regularly experience pain; 45% may experience daily pain [5]. Possible contributors to pain in SSc include arthralgia and arthritis, Raynaud’s phenomenon, odynophagia and digital ulcers [5]. Pain in SSc is associated with reduced health-related quality of life (HRQoL) and higher levels of depression [5].

Despite this, limited data describe opioid use in SSc. Oxycodone has been shown to be effective in managing pain related to severe digital ulcers and to improve adherence with wound care procedures [6]. However, there is increasing recognition of opioid-related harms, including misuse, dependence and overdose [4]. In SSc there may be additional, disease-specific risks of opioid use [7], including exacerbation of gastrointestinal (GI) symptoms and respiratory depression in those with pulmonary disease, despite noting its frequent use for management of end-stage dyspnoea. Given the limited data describing opioid use in SSc, we sought to describe the frequency and clinical associations of opioid use and to compare the impacts of opioid use in SSc.

## Methods

Participants were recruited from the Australian Scleroderma Cohort Study (ASCS), a multicentre Australian study of risk and prognostic factors in SSc from 16 SSc centres across seven states and territories around Australia. The study was approved by the human research ethics committees of participating sites (HREC-A 020/07). Written informed consent was obtained from all participants. All participants met ACR/EULAR criteria for SSc [8] and had a definable disease subclass according to the LeRoy criteria (dcSSc or lcSSc) [9]. Participants were reviewed annually, with demographic and disease data collected prospectively. Medication use was recorded at each visit as ‘current’, ‘previously’ or ‘never’. Opioid use was recorded as a medication class; the formulation, dosage or indication was not recorded. Study participants were grouped into those who had ever received opioids since recruitment (opioid-exposed group) and those who had not (opioid-naïve group). Current opioid users were those identified as currently using opioids at each visit. Of those with opioid use data at two or more consecutive visits, the proportion of those receiving opioids at two or more consecutive study visits was also recorded and was considered to be long-term opioid use in this study.

The 36-Item Short Form Health Survey (SF-36) pain scores were calculated using the average of two SF-36 questions relating to pain: ‘How much bodily pain have you had during the past 4 weeks?’ (‘none’, ‘very mild’, ‘mild’, ‘moderate’, ‘severe’ or ‘very severe’) and ‘During the past 4 weeks, how much did pain interfere with your normal work?’ (‘not at all’, ‘a little bit’, ‘moderately’, ‘quite a bit’ or ‘extremely’). Each item was then transformed into a score of 0–100 for analysis, with lower scores indicating higher levels of pain. Upper GI symptoms were defined as a previous history of Barrett’s oesophagus, gastric antral vascular ectasia, oesophageal dysmotility/strictures, dysphagia, reflux or vomiting. Lower GI symptoms included a history of bowel dysmotility, pseudo-obstruction, constipation, faecal incontinence, diarrhoea or bloating. Medsger severity scale (MSS) scores were calculated to assess the burden of SSc-related complications at each study visit [10]. Modified Charlson Comorbidity Index (CCI) scores were used to measure multimorbidity [11] (Supplementary Data S1, available at *Rheumatology Advances in Practice* online). The definitions of other clinical variables are described in Supplementary Data S1, available at *Rheumatology Advances in Practice* online.

Outcomes are presented as mean (s.d.) for normally distributed continuous variables, median [interquartile range (IQR)] for non-normally distributed continuous variable, and as number (percentage) for discrete variables. Between-group comparisons were performed using two-sample *t*-test, Wilcoxon rank sum test or chi-squared test as appropriate. Generalised estimating equations (GEEs) using an exchangeable correlation structure were used to model longitudinal data involving repeated measures. Covariates were chosen if they were either clinically relevant or statistically significant on univariate analysis (*P* < 0.05) and were not collinear. Results are presented as odds ratios (ORs) or regression coefficients (RCs) with accompanying 95% CIs. Analyses were performed using Stata 17.0 (StataCorp, College Station, TX, USA).

## Results

Of 1951 participants, 1673 (87.8%) were female, with a mean age at SSc onset of 46.7 years (s.d. 14.4) (Table 1). A total of 231 participants (11.8%) had ever received opioids, of whom 53 (22.9%) were on opioids at baseline. Overall, 1626 participants (83.3%) had data at two or more visits; 95 (45.7%) opioid users received opioids at two or more consecutive study visits. There were no differences in age at SSc onset, sex, race, SSc subtype or serology between groups, although opioid-exposed participants had a longer follow-up duration (*P* < 0.01). The opioid-exposed group had higher SSc severity (MSS scores, *P* < 0.01) and more multimorbidity (*P* < 0.01). This group also had lower (worse) baseline SF-36 pain scores (*P* < 0.01).

**Table 1.** Demographic data, SSc disease features and treatments

Characteristics	Opioid exposed^a^ [*n* = 231 (11.8%)]	Opioid naïve [*N* = 1720 (88.2%]	*P*-value^b^
Age at SSc onset, years, mean (s.d.)	45.3 (13.4)	46.9 (14.5)	0.12
Male, *n* (%)	37 (16.0)	241 (14.0)	0.41
Caucasian, *n* (%)	195 (89.9)	1482 (91.2)	0.37
Diffuse SSc, *n* (%)	66 (28.6)	455 (26.5)	0.49
MRSS (highest)^a^, median (IQR)	9 (5–17)	8 (5–16)	0.54
SSc duration at recruitment, years, median (IQR)	8.1 (2.4–17.9)	7.2 (2.6–15.6)	0.35
Follow-up, years, median (IQR)	7.2 (3.4–11.2)	3.9 (1.2–7.9)	<0.01
Smoker (current or previous)^c^, *n* (%)	151 (65.4)	822 (47.8)	<0.01
MSS score at baseline, median (IQR)	6 (4–8)	5 (3–7)	<0.01
Multimorbidity (Charlson Comorbidity Index ≥4)^c^	91 (39.4)	360 (20.9)	<0.01
ANA centromere, *n* (%)	111 (48.7)	764 (46.2)	0.48
ENA			
Scl-70, *n* (%)	33 (14.8)	244 (15.0)	0.94
Ro, *n* (%)	25 (11.2)	153 (9.4)	0.40
RNA polymerase-3, *n* (%)	28 (15.4)	150 (13.4)	0.48
SF-36 pain score at baseline^d^ (*n* = 1582), median (IQR)	45 (22.5–60)	67.5 (45–80)	<0.01
SF-36 pain scores lower (worse) than SSc cohort median (57.5 units) (*n* = 1620), *n* (%)	192 (89.7)	937 (66.6)	<0.01
SF-36 pain scores lower (worse) than general population mean of 50 units (*n* = 1620), *n* (%)	185 (86.5)	867 (61.7)	<0.01
PAH^c^, *n* (%)	20 (8.7)	162 (9.4)	0.71
ILD^b^^,^^c^, *n* (%)	86 (37.2)	458 (26.6)	<0.01
FVC <80%^c^ (*n* = 1876), *n* (%)	98 (44.1)	529 (32.0)	
DLCO <80%^c^ (*n* = 1735), *n* (%)	178 (86.0)	1203 (78.7)	0.02
Worsening dyspnoea in the last month^c^ (*n* = 1921), *n* (%)	145 (63.9)	731 (43.2)	<0.01
WHO functional class 3 or 4^c^ (*n* = 1885), *n* (%)	115 (50.7)	516 (31.1)	<0.01
RP^c^, *n* (%)	230 (99.6)	1704 (99.1)	0.45
Digital ulcers^c^, *n* (%)	145 (62.8)	887 (51.6)	<0.01
Non-hand cutaneous ulcers^c^ (*n* = 1388), *n* (%)	69 (30.9)	157 (13.5)	<0.01
Synovitis^c^, *n* (%)	132 (57.1)	656 (38.1)	<0.01
Tendon friction rubs^c^ (*n* = 1920), *n* (%)	22 (9.6)	150 (8.9)	0.71
Joint contracture^c^ (*n* = 1925), *n* (%)	116 (50.7)	672 (39.6)	<0.01
Complicated calcinosis^c^ (*n* = 1229), *n* (%)	25 (11.9)	109 (10.7)	0.63
Proximal weakness (MMT <5/5)^c^ (*n* = 1910), *n* (%)	71 (31.0)	337 (20.1)	<0.01
Upper GI symptoms^c^^,^^e^, *n* (%)	224 (97.0)	1517 (88.2)	<0.01
Lower GI symptoms^c^^,^^f^, *n* (%)	207 (89.6)	1379 (80.2)	<0.01
Malignancy^c^, *n* (%)	72 (31.2)	382 (22.2)	<0.01
NSAID use^c^, *n* (%)	131 (56.7)	551 (32.0)	<0.01
Calcium channel blocker^c^, *n* (%)	179 (77.5)	1103 (64.1)	<0.01
Iloprost infusion^c^, *n* (%)	57 (24.7)	226 (13.2)	<0.01
Non-corticosteroid immunosuppression^c^^,^^g^, *n* (%)	137 (59.3)	787 (45.8)	<0.01
Prednisolone^c^, *n* (%)	149 (64.5)	749 (43.6)	<0.01

a Opioid exposure defined as any recorded opioid use from recruitment.

b ILD diagnosed on high-resolution CT.

c Denotes ever recorded from SSc onset.

d If baseline data was not available, earliest data from first three study visits was used.

e Upper GI symptoms include history of Barret’s oesophagus, GAVE, oesophageal dysmotility, oesophageal strictures, dysphagia, reflux or vomiting.

f Lower GI symptoms include history of bowel dysmotility, pseudo-obstruction, constipation, faecal incontinence, diarrhoea or bloating.

g Immunosuppressive treatment defined as ever receiving corticosteroids or synthetic or biologic DMARDs.

ACE: angiotensin-converting enzyme; DLCO: diffusing capacity for carbon monoxide; ENA: extractable nuclear antigen; IHD: ischaemic heart disease; LVEF: left ventricular ejection fraction; PROMIS: Patient-Reported Outcome Measures Information System; RV: right ventricular; RVSP: right ventricular systolic pressure; SRC: scleroderma renal crisis.

Cardiopulmonary disease including interstitial lung disease (ILD), impaired spirometry and worsening dyspnoea (*P* < 0.05) were more frequent in opioid-exposed participants, although pulmonary arterial hypertension (PAH) was not (*P* = 0.71). Opioid-exposed participants were more likely to have reported World Health Organization (WHO) class III/IV dyspnoea and a history of malignancy (*P* < 0.01). Multiple musculoskeletal disease features including digital and cutaneous ulceration, synovitis, joint contractures and proximal muscle weakness were more common in opioid-exposed participants (*P* < 0.01). Upper and lower GI symptoms were more common in this group (*P* < 0.01). Use of NSAIDs, calcium channel blockers, iloprost infusion, corticosteroids and non-corticosteroid immunosuppression was more common in opioid-exposed participants (*P* < 0.01).

### Associations of current opioid use at each study visit

Using GEEs to model SF-36 pain scores over time, current opioid use at each visit was associated with a 3.8-unit worsening in pain scores [RC −3.8 units (95% CI −7.3, −0.2), *P* = 0.04]. A multivariable model of the associations of current opioid use at each visit identified that increasing age [OR 1.2 (95% CI 1.1, 1.3), *P* < 0.01], low SF-36 physical component summary (PCS) scores [OR 1.8 (95% CI 1.3, 2.4), *P* < 0.01] and SF-36 mental component summary (MCS) scores [OR 1.8 95% CI 1.1, 3.0), *P* = 0.02] were all associated with an increased likelihood of current opioid use at each study visit, although sex was not (Table 2; univariable analysis in Supplementary Table S1, available at *Rheumatology Advances in Practice* online). An increasing modified Rodnan skin score (MRSS) was associated with a lower likelihood of current opioid use [OR 0.8 (95% CI 0.7, 0.9), *P* < 0.01]. Digital ulcers [OR 1.5 (95% CI 1.1, 2.0), *P* = 0.01] and synovitis [OR 1.5 (95% CI 1.1, 2.1), *P* = 0.02] were associated with current opioid use, although joint contractures were not. Lower GI tract symptoms [OR 1.8 (95% CI 1.3, 2.6), *P* < 0.01] were associated with an increased frequency of current opioid use. Forced vital capacity (FVC) <80% was associated with current opioid use [OR 1.5 (95% CI 1.0, 2.1), *P* = 0.04], although a patient-reported increase in dyspnoea was not.

**Table 2.** Multivariable model using GEE regression of associations of current opioid use at each study visit (*N* = 5152 observations)

Variable at each study visit	OR	95% CI	*P*-value
Age at each review (5-year increments)	1.2	1.1, 1.3	<0.01
Male sex	1.3	0.7, 2.3	0.44
MRSS (5-point increment)	0.8	0.7, 0.9	<0.01
SF-36 MCS score <50^a^	1.8	1.1, 3.0	0.02
SF-36 PCS score <50^a^	1.8	1.3, 2.4	<0.01
Synovitis	1.5	1.1, 2.1	0.02
Digital ulcers	1.5	1.1, 2.0	0.01
Joint contractures	1.2	0.9, 1.7	0.24
Upper GI symptoms	1.2	0.8, 1.7	0.38
Lower GI symptoms^b^	1.8	1.3, 2.6	<0.01
Worsening dyspnoea at each study visit^c^	1.2	0.9, 1.6	0.28
FVC <80%	1.5	1.0, 2.1	0.04

a Standardised SF-36 MCS and PCS scores used, where scores <50 indicate poorer mental or physical health than the general population median.

b Lower GI symptoms include history of bowel dysmotility, pseudo-obstruction, constipation, faecal incontinence, diarrhoea or bloating.

c Patient-reported worsening of dyspnoea (yes/no) in the month prior to the study visit.

### HR-QoL and physical function

Using multivariable GEE regression (adjusting for age, sex, MRSS and SF-36 bodily pain scores; Supplementary Table S2, available at *Rheumatology Advances in Practice* online), current opioid use was associated with worse dyspnoea as measured by FACIT-Dyspnoea scores [RC 2.0 units (95% CI 0.8, 3.1), *P* < 0.01] and higher functional disability as measured by HAQ-DI scores [RC 0.2 (95% CI 0.2, 0.3), *P* < 0.01]. Current opioid use was associated with poorer mental well-being as measured by PROMIS-29 anxiety [RC 2.5 (95% CI 0.9, 4.0), *P* < 0.01] and depression scores [RC 2.5 (95% CI 0.9, 4.0), *P* < 0.01]. Current opioid use was also associated with a higher burden of fatigue as measured by FACIT fatigue scores [RC 3.0 (95% CI 1.2, 4.8), *P* < 0.01].

## Discussion

In a large Australian SSc cohort, 11.8% of participants had ever used opioids, with use at multiple consecutive study visits observed in 4.9% of participants. We were unable to provide more granular detail around type, indication and duration of opioid use, as these data are not captured by the ASCS. However, we identified important associations of opioid use in SSc, including musculoskeletal, GI and lung involvement. Opioid prescription was associated with poorer QoL and physical function at each study visit.

Limited data describe the frequency of opioid prescription in other SSc cohorts. In one smaller cross-sectional study, 16% of patients used opioids [12], while in another study of SSc patients with chronic pain, 5.2% used strong opioids and 10.4% used mild opioids [13]. The use of opioids in SSc appears less frequent than is reported in SLE or inflammatory arthritis, where 12–23% [2] and 30–40% [3] of patients, respectively, have been reported to be prescribed opioids. This is notable because our data suggest a similar burden of chronic pain in SSc compared with inflammatory arthritis. Our cohort reported a significant burden of pain; 60–80% had more pain than the general Australian population [14]. Other studies have also reported a high frequency of pain in SSc, with daily pain affecting 45% of people with SSc [5] compared with ≈40–50% of those with RA or PsA [15, 16]. The variation in use of opioids in these patient populations may relate to SSc disease-specific risks of opioid analgesia, including worsening GI symptoms or respiratory depression in those with cardiopulmonary complications [7], as well as attendant risks of polypharmacy. Use of non-opioid and non-pharmacological analgesia other than NSAIDs is not captured in the ASCS, thus could not be accounted for in this study. As the long-term risks of opioid analgesia are well-recognised by rheumatologists [4, 7], it is possible that multiple other analgesic regimens utilised in preference to opioids are not captured by the ASCS.

Non-SSc conditions such as lower back pain and osteoarthritis may contribute to pain in SSc [13]. While the ASCS does not capture non-SSc-specific causes of pain, painful musculoskeletal conditions including arthritis and lower back pain may increase with age. Accordingly, a higher burden of musculoskeletal pain may explain our observed association between current opioid use and increasing age. Gastrointestinal symptoms were also more common in opioid users. While opioids or opioid derivatives may be prescribed in chronic idiopathic diarrhoea [17], opioid analgesia is not routinely recommended to manage GI complications in SSc [18]. Patients prescribed opioids may instead be predisposed to develop GI symptoms, lending support to the idea that opioid use exacerbates SSc-associated GI disturbance. Moreover, respiratory disease was associated with opioid use in our cohort, possibly because opioids may be trialled in these patients to manage dyspnoea or cough. Our group has shown that severe breathlessness is present in almost one in four people living with SSc and that symptoms (e.g. pain and dyspnoea) often occur concurrently [19]. Further controlled data exploring the efficacy of opioids, and other options, in chronic breathlessness due to ILD and PAH in both general and SSc cohorts are required.

Our data demonstrate consistently worse HRQoL in those prescribed opioid analgesia. Current opioid use was associated with universally poorer patient- and physician-reported measures of physical function, disability and fatigue, even after adjusting for comorbid pain. Furthermore, patient-reported anxiety, depression and mental health scores were universally worse in those receiving opioids. Pain undoubtedly contributes to poorer psychological well-being. These data may suggest that opioid analgesia cannot negate the significant symptom burden of chronic pain and its contribution to reduced HRQoL, physical function and mental health in SSc. However, this may also indicate increased reporting of pain or pain amplification in those with poorer psychological well-being, in keeping with data in other conditions associated with musculoskeletal pain showing a relationship between higher levels of chronic pain and depression [20].

A major limitation of this study was the lack of data regarding type, indication and duration of opioid prescriptions. The ASCS protocol does not collect specific drug types, dosages or indications for prescription, inherently limiting any analysis that examines medication use. Furthermore, because data are collected annually, it was difficult to quantify the duration of opioid use, and we were only able to measure the number of consecutive visits at which a person used opioids as a surrogate marker of longer-term opioid use. For example, those using intermittent immediate-release opioids to manage digital ulceration at a similar time each year may have been identified as ‘current’ opioid users at each review and thus counted in this study as those who used opioids at consecutive visits. These participants are likely to have clinical differences compared with those individuals who were prescribed long-term extended-release opioids for management of other comorbidities such as chronic pain. Finally, in these observational data there is a degree of confounding bias associated with any interpretation of treatment data, therefore only cautious conclusions can be drawn about the associations between clinical manifestations and treatment. However, given the paucity of opioid use data in SSc, this large, longitudinal SSc cohort can be used to generate hypotheses about the potential correlates and impact of opioid use in SSc.

## Conclusion

In 1951 individuals with SSc, 11.8% had used opioids, with 4.9% using opioids at multiple consecutive annual study visits. Painful musculoskeletal disease features, digital ulceration, lower GI symptoms and impaired FVC were associated with current opioid use. Opioid-exposed participants experienced a higher symptom burden and poorer HRQoL and physical function.

## Supplementary Material

rkae144_Supplementary_Data

## Data Availability

Data are available upon reasonable request.
