# Supplementary material for: The frequency and clinical associations of opioid use in systemic sclerosis
Source: Rheumatol Adv Pract. 2024 Nov 15;8(4):rkae144. doi: 10.1093/rap/rkae144 (PMC11634672; doi:10.1093/rap/rkae144)
Supplement: rkae144_Supplementary_Data [file rkae144_supplementary_data.docx]

**Supplementary Table S1: Univariable GEE Regression modelling exploring associations of current opioid use at each visit.**

|  | **N^** | **OR** | **95% CI** | **p** |
| --- | --- | --- | --- | --- |
| **Disease features and associations of opioid use** | | | | |
| Age at SSc onset | 9784 | 0.99 | 0.98-1.00 | 0.04 |
| Male sex | 10147 | 1.26 | 0.80-1.97 | 0.32 |
| Modified Rodnan Skin Score | 8584 | 0.96 | 0.94-0.98 | <0.01 |
| SF-36 MCS Score <50 | 6104 | 1.79 | 1.37-2.34 | <0.01 |
| SF-36 PCS Score <50 | 6104 | 2.39 | 1.56-3.67 | <0.01 |
| Synovitis | 9904 | 1.59 | 1.24-2.03 | <0.01 |
| Digital ulcers | 9541 | 1.28 | 1.03-1.59 | 0.02 |
| Joint contractures | 8813 | 1.30 | 1.02-1.67 | 0.04 |
| Upper gastrointestinal symptoms^1^ | 9334 | 1.24 | 0.98-1.57 | 0.07 |
| Lower gastrointestinal symptoms^2^ | 9299 | 1.83 | 1.45-2.30 | <0.01 |
| FVC<80% | 9464 | 1.48 | 1.14-1.91 | <0.01 |
| Pulmonary arterial hypertension | 10417 | 1.47 | 0.95-2.28 | 0.08 |
| Interstitial lung disease^3^ | 3342 | 1.42 | 0.82-2.46 | 0.21 |
| **Impact of opioid use on patient-reported outcome measures of physical function and HRQoL** | | | | |
| PROMIS-29 Function^^^ | 2747 | -3.42 units | -4.65 to -2.20 | <0.01 |
| PROMIS-29 Fatigue^^^ | 2805 | 2.41 units | 0.98 to 3.83 | <0.01 |
| PROMIS-29 Depression^^^ | 2748 | 3.10 units | 1.65-4.55 | <0.01 |
| PROMIS-29 Anxiety^^^ | 2713 | 2.81 units | 1.34-4.29 | <0.01 |
| HAQ-DI score | 5901 | 0.24 units | 0.17 to 0.30 | <0.01 |
| FACIT Dyspnoea | 2628 | 1.78 units | 0.73 to 2.83 | <0.01 |
| FACIT Fatigue | 2768 | 6.06 units | 3.92 to 8.21 | <0.01 |

Abbreviations: FACIT (functional assessment of chronic illness therapy survey), GEE (generalised estimating equations), HAQ-DI (health assessment questionnaire disability index), HRQoL (health related quality of life), m (metre), MRSS (modified Rodnan skin score), PAH (pulmonary arterial hypertension), PROMIS (patient-reported outcome measures information system), SF-36 (Short Form Survey-36). ^1^Upper gastrointestinal symptoms include dysphagia, reflux or vomiting. ^2^Lower gastrointestinal symptoms includes history of bowel dysmotility, pseudo-obstruction, constipation, faecal incontinence, diarrhoea or bloating. ^3^ILD diagnosed on high-resolution computed tomography (HRCT). ^Standardised score.

**Supplementary Table S2: GEE Regression modelling describing the impact of current opioid use on HRQoL Scores (adjusted for age at each review, sex, MRSS and SF-36 bodily pain scores)**

|  | N^ | Regression coefficient | 95% Confidence Interval | p-value |
| --- | --- | --- | --- | --- |
| HAQ-DI score | 5242 | 0.21 units | 0.15 to 0.27 | <0.01 |
| FACIT Dyspnoea | 2235 | 1.97 units | 0.84 to 3.10 | <0.01 |
| FACIT-Fatigue* | 2338 | 2.99 units | 1.15 to 4.82 | <0.01 |
| PROMIS-29 Depression^ | 2336 | 2.46 units | 0.94 to 3.97 | <0.01 |
| PROMIS-29 Anxiety^ | 2311 | 2.46 units | 0.90 to 4.02 | <0.01 |

Abbreviations: FACIT (functional assessment of chronic illness therapy survey), GEE (generalised estimating equation), HAQ-DI (health assessment questionnaire disability index), m (metre), MRSS (modified Rodnan skin score), PAH (pulmonary arterial hypertension), PROMIS (patient-reported outcome measures information system), SF-36 (Short Form Survey-36). *Score inverted for ease of comprehension as more negative score indicates higher symptom burden. ^Standardised score.

**Supplementary Data S1. Definition and collection of clinical variables.**

Disease manifestations and autoantibody results were considered present if ever reported from SSc onset, defined as the first non-Raynaud phenomenon SSc manifestation. Clinical assessment was performed annually by the study physician to assess the presence of SSc symptoms and record examination findings, including proximal muscle atrophy and weakness (defined as power <5/5 on manual muscle testing). Ischaemic heart disease (IHD) was defined as patient-reported angina or myocardial infarction, or abnormal coronary angiogram. Participants were screened annually for pulmonary arterial hypertension (PAH) and interstitial lung disease (ILD) with annual screening pulmonary function tests (PFTs), including measurement of percent-predicted forced vital capacity (FVC) and diffusing capacity for carbon monoxide (DLCO, corrected for haemoglobin) and transthoracic echocardiography. PAH was defined as participants with a RHC performed which met revised^18^ or previous^19^ PAH classification criteria. ILD was diagnosed using high-resolution computed tomography (HRCT) of the chest performed at physician discretion in response to clinical assessment or abnormal PFTs. Myositis was defined by a positive muscle biopsy and muscle atrophy was recorded at each visit by the treating clinician. Proximal weakness was defined as power on manual muscle testing (MMT) results <5/5 by the treating clinician. Upper gastrointestinal symptoms were defined as a history of Barrett’s oesophagus, gastric antral vascular ectasia, oesophageal dysmotility/stricture, dysphagia, reflux or vomiting, while lower gastrointestinal symptoms were defined as history of bowel dysmotility or pseudo-obstruction, constipation, faecal incontinence, diarrhoea, or bloating. Medsger Severity Scores (MSS) were calculated to assess burden of SSc-related complications at each study visit^20^. To measure multimorbidity, we calculated a modified Charlson Comorbidity Index (CCI) score^21^. A list of included items is provided in Supplementary Table S3; data for some variables (including hemiplegia, HIV/AIDS and dementia) were excluded as these data are not collected as part of the ASCS protocol. A CCI score≥4 was defined as a significant comorbidity burden^22^, with the highest available score being 19.

World Health Organisation (WHO) Functional Class breathlessness was recorded at each visit. Participants were asked at each visit if they had been more breathless in the last month (yes/no). Patient-reported outcome measures (PROMs) were collected at each visit, including the Health Assessment Questionnaire-Disability Index (HAQ-DI), Short Form-36 Survey (SF-36), Functional Assessment of Chronic Illness Therapy (FACIT) and PRO Measures Information System (PROMIS)-29.

**Supplementary Table S3: CCI Score calculation**

| **Original Charlson Comorbidity Index** | | **Adaptation** | |
| --- | --- | --- | --- |
| Item | Score | Item | Score |
| Cerebrovascular disease | 1 | Patient-reported Stroke/TIA* | 1 |
| Congestive heart failure | 1 | LVEF≤50%* | 1 |
| COPD/Asthma | 1 | Patient-reported COPD or asthma | 1 |
| Dementia | 1 | Not recorded; excluded | N/A |
| Depression | 1 | Not recorded; excluded | N/A |
| Hypertension | 1 | Patient-reported hypertension* | 1 |
| Diabetes without end organ dysfunction | 1 | Patient-reported diabetes* | 1 |
| Diabetes with end organ damage | 2 | Not recorded; excluded | N/A |
| Liver disease - Mild | 1 | Not recorded; excluded | N/A |
| Liver disease – moderate or severe | 3 | Not recorded; excluded | N/A |
| Myocardial infarction | 1 | Patient-reported angina or myocardial infarction* | 1 |
| Peripheral vascular disease | 1 | Patient-reported peripheral vascular disease or treatments* | 1 |
| Rheumatic disease | 1 | Applicable to all patients with SSc | 1 |
| Peptic ulcer disease | 1 | Excluded; not recorded independently of other gastrointestinal SSc manifestations | N/A |
| Hemiplegia | 2 | Not recorded; excluded | N/A |
| Moderate to severe renal disease | 2 | Creatinine>265umol/L ever, or previous dialysis or renal transplantation* | 2 |
| Any tumour | 2 | Patient-reported malignancy (excluding NMSC) | 2 |
| Metastatic solid tumour | 6 | Not recorded; excluded | N/A |
| Skin ulcers or cellulitis | 2 | Not recorded; excluded | N/A |
| Takes warfarin | 1 | Warfarin or other anticoagulation | 1 |
| Leukaemia | 2 | Patient-reported leukaemia | 2 |
| Lymphoma | 2 | Patient-reported lymphoma | 2 |
| HIV/AIDS | 6 | Not recorded; excluded | N/A |
| **Maximum score** | **38** | **Maximum Score** | **19** |

Abbreviations: AIDS (acquired immunodeficiency syndrome), COPD (chronic obstructive pulmonary disease), HIV (human immunodeficiency virus), LVEF (left ventricular ejection fraction), NMSC (non-melanoma skin cancer), SSc (systemic sclerosis), TIA (transient ischaemic attack), umol/L (micromoles per litre)
